# Supplementary material for: Acquisition of fungi from the environment modifies ambrosia beetle mycobiome during invasion
Source: PeerJ. 2019 Nov 18;7:e8103. doi: 10.7717/peerj.8103 (PMC6870512; doi:10.7717/peerj.8103)
Supplement: Figure S1 — Blue dots indicate old-growth forests, red dots indicate restored forests. All sites were located in north-eastern Italy. Each site is identified by the name of the locality. Map Data 2019 Google. [file peerj-07-8103-s001.docx]

**Acquisition of fungi from the environment modifies ambrosia beetle mycobiome during invasion**

Davide Rassati, Lorenzo Marini, Antonino Malacrinò

**Fig. S1:** Map showing the geographical position of the 10 forest sites where individuals of the ambrosia beetles *X. germanus* and *X. saxesenii* were collected. Blue dots indicate old-growth forests, red dots indicate restored forests. All sites were located in north-eastern Italy. Each site is identified by the name of the locality. Map Data @ 2019 Google

**
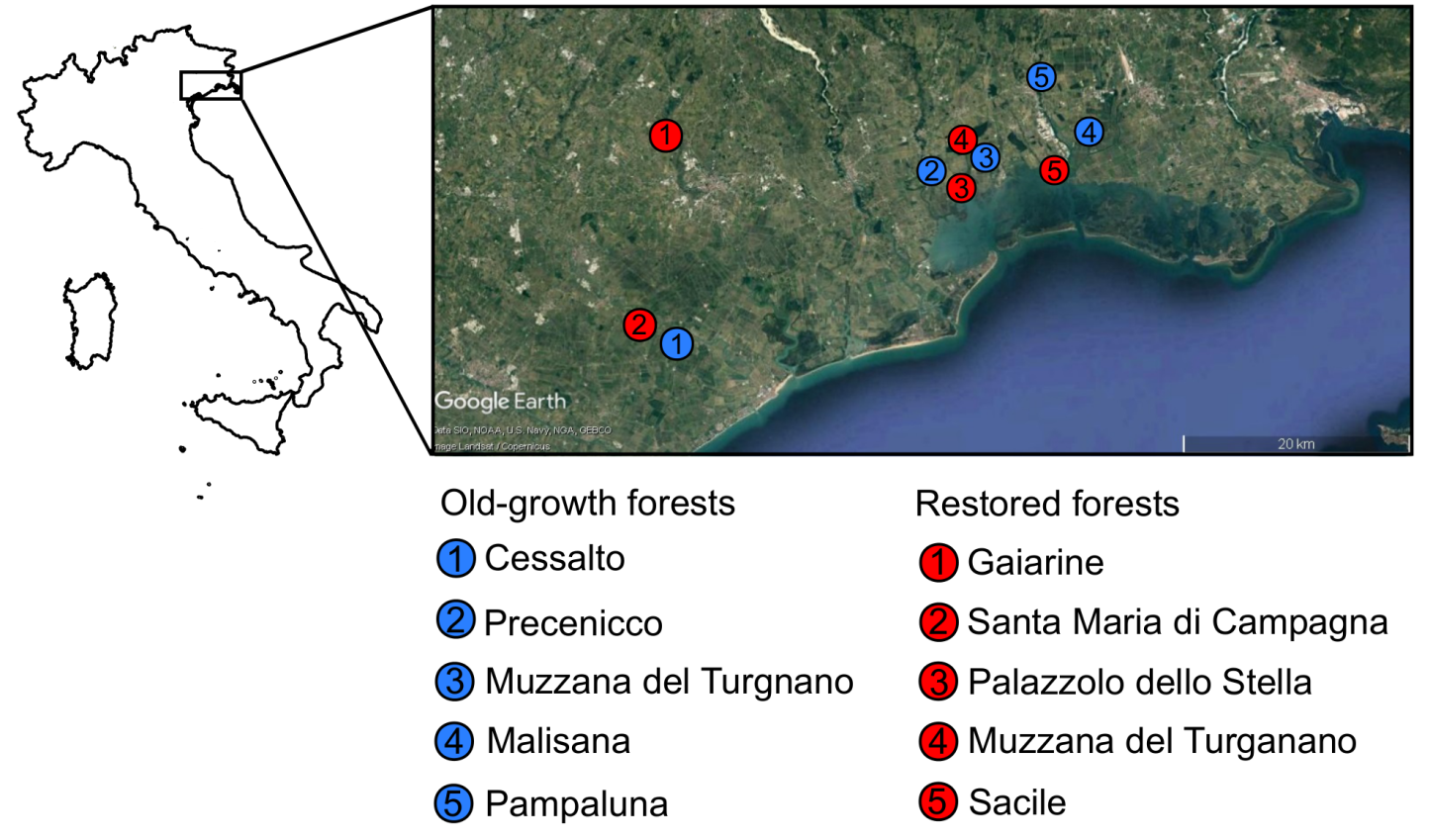
**
