# Supplementary material for: Acquisition of fungi from the environment modifies ambrosia beetle mycobiome during invasion
Source: PeerJ. 2019 Nov 18;7:e8103. doi: 10.7717/peerj.8103 (PMC6870512; doi:10.7717/peerj.8103)
Supplement: Table S1 — The main parameters were measured at each site in two circular areas (15 m radius) located close to the boundary of the point where trap was placed. [file peerj-07-8103-s002.docx]

**Acquisition of fungi from the environment modifies ambrosia beetle mycobiome during invasion**

Davide Rassati, Lorenzo Marini, Antonino Malacrinò

**Table S1: Forest type (old-growth vs restored), geographic coordinates, area (ha), Shannon Index (*H*), and mean tree diameter (DBH, cm) for each of the 10 forest sites where ambrosia beetle individuals were collected**. The main parameters were measured at each site in two circular areas (15m radius) located close to the boundary of the point where trap was placed.

| **Site** | **Forest type** | **Latitude** | **Longitude** | **Area** | ***H*** | **DBH** | |
| --- | --- | --- | --- | --- | --- | --- | --- |
| Avronchi | Old-growth | 45.79178 | 13.12092 | 165.15 | 0.57 | 25.58 |  |
| Bando | Old-growth | 45.77686 | 13.06389 | 9.140 | 1.80 | 30.27 |  |
| Cessalto | Old-growth | 45.70094 | 12.61773 | 28.84 | 1.64 | 23.98 |  |
| Malisana | Old-growth | 45.80601 | 13.24031 | 23.23 | 0.99 | 24.88 |  |
| Pampaluna | Old-growth | 45.8538 | 13.19542 | 12.15 | 1.34 | 28.70 |  |
| Brussa | Restored | 45.76713 | 13.07946 | 32.91 | 0.71 | 26.73 |  |
| Muzzana | Restored | 45.79886 | 13.10984 | 2.37 | 1.58 | 23.27 |  |
| Otello | Restored | 45.86135 | 12.49367 | 2.61 | 2.27 | 20.78 |  |
| Sacile | Restored | 45.78189 | 13.20181 | 5.22 | 1.61 | 26.69 |  |
| San Marco | Restored | 45.70533 | 12.57947 | 16.36 | 2.21 | 23.73 |  |
