# Supplementary material for: Acquisition of fungi from the environment modifies ambrosia beetle mycobiome during invasion
Source: PeerJ. 2019 Nov 18;7:e8103. doi: 10.7717/peerj.8103 (PMC6870512; doi:10.7717/peerj.8103)
Supplement: Table S2 — For each sampling site. P-value as adjusted for multiple comparisons using FDR method. *, number of samples after filtering [file peerj-07-8103-s003.docx]

**Acquisition of fungi from the environment modifies ambrosia beetle mycobiome during invasion**

Davide Rassati, Lorenzo Marini, Antonino Malacrinò

**Table S2: Multivariate analysis comparing the fungal communities associated with the exotic ambrosia beetle *X. germanus* and the native ambrosia beetle *X. saxesenii***. For each sampling site. *P*-value as adjusted for multiple comparisons using FDR method. * = number of samples after filtering

| **Site** | **Forest type** | ***X. germanus* (n)*** | ***X. saxesenii* (n)*** | **F** | ***P*_adj_** |
| --- | --- | --- | --- | --- | --- |
| Avronchi | Old-growth | 14 | 4 | 4.07 | 0.002 |
| Bando | Old-growth | 13 | 8 | 5.31 | 0.001 |
| Cessalto | Old-growth | 15 | 12 | 5.52 | 0.001 |
| Malisana | Old-growth | 8 | 13 | 2.55 | 0.001 |
| Pampaluna | Old-growth | 15 | 9 | 4.9 | 0.001 |
| Brussa | Restored | 5 | 15 | 1.47 | 0.01 |
| Muzzana | Restored | 9 | 10 | 1.39 | 0.004 |
| Otello | Restored | 14 | 13 | 1.63 | 0.001 |
| Sacile | Restored | 9 | 9 | 1.52 | 0.002 |
| San Marco | Restored | 9 | 9 | 1.69 | 0.001 |
