# Supplementary material for: Acquisition of fungi from the environment modifies ambrosia beetle mycobiome during invasion
Source: PeerJ. 2019 Nov 18;7:e8103. doi: 10.7717/peerj.8103 (PMC6870512; doi:10.7717/peerj.8103)
Supplement: Table S3 — The guild column represents the prediction of the functional role of each genus, according to the FUNGuild database and manually curated. [file peerj-07-8103-s004.docx]

**Acquisition of fungi from the environment modifies ambrosia beetle mycobiome during invasion**

Davide Rassati, Lorenzo Marini, Antonino Malacrinò

**Table S3: Relative abundance of each fungal genus found associated with the exotic *X. germanus* and the native *X. saxesenii*.** The guild column represents the prediction of the functional role of each genus, according to the FUNGuild database and manually curated.

| **Genus** | ***X. germanus*** | ***X. saxesenii*** | **Guild** |
| --- | --- | --- | --- |
| *Acremonium* | 0.04% | 0.05% | Saprotroph |
| *Alternaria* | 0.48% | 0.12% | Plant Pathogen |
| *Ambrosiella* | 2.25% | 0.00% | Mutualist |
| *Angustimassarina* | 0.02% | 0.00% | Saprotroph |
| *Apodus* | 0.02% | 0.00% | Saprotroph |
| *Arthrobotrys* | 0.04% | 0.00% | Saprotroph |
| *Aspergillus* | 0.35% | 0.39% | Plant Pathogen |
| *Aureobasidium* | 0.66% | 0.60% | Endophyte |
| *Beauveria* | 0.00% | 0.02% | Entomopathogen |
| *Blumeria* | 0.04% | 0.00% | Plant Pathogen |
| *Botryosphaeria* | 0.42% | 0.11% | Plant Pathogen |
| *Cadophora* | 0.07% | 0.00% | Endophyte |
| *Candida* | 0.00% | 0.08% | Yeast |
| *Chaetomium* | 0.00% | 0.02% | Saprotroph |
| *Cheiromyces* | 0.02% | 0.00% | Saprotroph |
| *Chloridium* | 0.04% | 0.00% | Ectomycorrhizal |
| *Choiromyces* | 0.00% | 0.00% | Ectomycorrhizal |
| *Cladosporium* | 1.52% | 2.65% | Plant Pathogen |
| *Claroideoglomus* | 0.02% | 0.00% | Arbuscular Mycorrhizal |
| *Clonostachys* | 0.06% | 0.00% | Plant Pathogen |
| *Colletotrichum* | 0.34% | 0.25% | Plant Pathogen |
| *Cryptococcus* | 0.18% | 0.02% | Saprotroph |
| *Cryptomarasmius* | 0.21% | 0.00% | Saprotroph |
| *Cryptosphaeria* | 0.15% | 0.00% | Plant Pathogen |
| *Cyberlindnera* | 0.00% | 0.01% | Yeast |
| *Cyphellophora* | 0.00% | 0.06% | Saprotroph |
| *Cystobasidium* | 0.01% | 0.00% | Yeast |
| *Didymella* | 0.00% | 0.00% | Plant Pathogen |
| *Drechmeria* | 0.00% | 0.00% | Animal Pathogen |
| *Epicoccum* | 0.00% | 0.00% | Plant Pathogen |
| *Erysiphe* | 0.00% | 0.02% | Plant Pathogen |
| *Eucasphaeria* | 0.01% | 0.00% | Saprotroph |
| *Eutypa* | 0.13% | 0.05% | Plant Pathogen |
| *Filobasidium* | 0.04% | 0.06% | Saprotroph |
| *Fusarium* | 0.15% | 0.29% | Plant Pathogen |
| *Fuscoporia* | 0.00% | 0.00% | Saprotroph |
| *Geosmithia* | 0.00% | 0.00% | Plant Pathogen |
| *Gibellulopsis* | 0.03% | 0.46% | Plant Pathogen |
| *Glomus* | 0.11% | 0.40% | Arbuscular Mycorrhizal |
| *Helvella* | 0.01% | 0.00% | Ectomycorrhizal |
| *Humicola* | 0.00% | 0.00% | Saprotroph |
| *Knufia* | 0.00% | 0.11% | Saprotroph |
| *Leptosphaeria* | 0.00% | 0.00% | Plant Pathogen |
| *Lophodermium* | 0.54% | 0.48% | Plant Pathogen |
| *Malassezia* | 0.03% | 0.12% | Yeast |
| *Massarina* | 0.00% | 0.18% | Saprotroph |
| *Melanconiella* | 0.18% | 0.00% | Saprotroph |
| *Metarhizium* | 0.00% | 0.00% | Enthomopathogen |
| *Meyerozyma* | 4.74% | 15.23% | Yeast |
| *Mortierella* | 0.19% | 0.31% | Saprotroph |
| *Mucor* | 0.10% | 0.30% | Saprotroph |
| *Mycocalicium* | 0.00% | 0.00% | Lichen Parasite |
| *Nothophoma* | 0.00% | 0.00% | Saprotroph |
| *Olpidium* | 0.00% | 0.03% | Plant Pathogen |
| *Papiliotrema* | 0.14% | 0.02% | Yeast |
| *Paraconiothyrium* | 0.18% | 0.00% | Saprotroph |
| *Paurocotylis* | 0.02% | 0.00% | Ectomycorrhizal |
| *Penicillium* | 0.18% | 1.49% | Saprotroph |
| *Periconia* | 0.08% | 0.00% | Plant Pathogen |
| *Pezizella* | 0.00% | 0.00% | Lichen Parasite |
| *Phaeoacremonium* | 0.00% | 0.00% | Plant Pathogen |
| *Phoma* | 0.03% | 0.03% | Plant Pathogen |
| *Plectosphaerella* | 0.00% | 0.00% | Plant Pathogen |
| *Plenodomus* | 0.28% | 0.01% | Saprotroph |
| *Pleotrichocladium* | 0.00% | 0.00% | Saprotroph |
| *Podospora* | 0.11% | 0.08% | Saprotroph |
| *Quambalaria* | 0.00% | 0.00% | Plant Pathogen |
| *Raffaelea* | 0.00% | 0.01% | Mutualist |
| *Rhizophlyctis* | 0.00% | 0.03% | Saprotroph |
| *Rhodotorula* | 0.01% | 0.01% | Yeast |
| *Rutstroemia* | 0.14% | 0.00% | Saprotroph |
| *Saccharomyces* | 0.51% | 0.54% | Saprotroph |
| *Saccharomycopsis* | 0.00% | 0.00% | Yeast |
| *Saitozyma* | 0.00% | 0.00% | Yeast |
| *Sarocladium* | 0.27% | 0.43% | Saprotroph |
| *Scedosporium* | 0.01% | 0.00% | Saprotroph |
| *Schizopora* | 0.00% | 0.00% | Saprotroph |
| *Schizosaccharomyces* | 0.05% | 0.00% | Yeast |
| *Sclerotinia* | 0.41% | 0.05% | Plant Pathogen |
| *Symmetrospora* | 0.00% | 0.08% | Yeast |
| *Talaromyces* | 0.00% | 0.43% | Saprotroph |
| *Taphrina* | 0.00% | 0.07% | Plant Pathogen |
| *Trichoderma* | 0.01% | 0.07% | Endophyte |
| *Unidentified* | 83.97% | 73.81% | Unidentified |
| *Vanrija* | 0.37% | 0.42% | Yeast |
| *Vermispora* | 0.00% | 0.00% | Animal Pathogen |
| *Volutella* | 0.01% | 0.00% | Plant Pathogen |
