# Supplementary material for: Acquisition of fungi from the environment modifies ambrosia beetle mycobiome during invasion
Source: PeerJ. 2019 Nov 18;7:e8103. doi: 10.7717/peerj.8103 (PMC6870512; doi:10.7717/peerj.8103)
Supplement: Table S4 [file peerj-07-8103-s005.docx]

**Acquisition of fungi from the environment modifies ambrosia beetle mycobiome during invasion**

Davide Rassati, Lorenzo Marini, Antonino Malacrinò

**Table S4:** **Results from the mixed-effects models testing the effect of beetle species and forest type on Chao1, Faith’s phylogenetic diversity and 1-Simpson indexes.**

|  | **Chao 1** | | **Phylogenetic diversity** | | **1-Simpson** | |
| --- | --- | --- | --- | --- | --- | --- |
| **Factor** | Χ^2^ | P | Χ^2^ | P | Χ^2^ | P |
| *Beetle species* | 72.29 | <0.001 | 173.74 | <0.001 | 135.20 | <0.001 |
| *Forest type* | 25.06 | <0.001 | 34.47 | <0.001 | 2.37 | 0.12 |
| *Beetle species x Forest type* | 111.53 | <0.001 | 199.45 | <0.001 | 19.20 | <0.001 |

df = 1
